# Bio-AnswerFinder: a system to find answers to questions from biomedical texts

**DOI:** 10.1093/database/baz137

**Published:** 2020-01-10

**Authors:** Ibrahim Burak Ozyurt, Anita Bandrowski, Jeffrey S Grethe

**Affiliations:** Center for Research in Biological Systems, University of California, San Diego, 9500 Gilman Drive, M/C 0608, La Jolla, CA 92093-0608

## Abstract

The ever accelerating pace of biomedical research results in corresponding acceleration in the volume of biomedical literature created. Since new research builds upon existing knowledge, the rate of increase in the available knowledge encoded in biomedical literature makes the easy access to that implicit knowledge more vital over time. Toward the goal of making implicit knowledge in the biomedical literature easily accessible to biomedical researchers, we introduce a question answering system called Bio-AnswerFinder. Bio-AnswerFinder uses a weighted-relaxed word mover's distance based similarity on word/phrase embeddings learned from PubMed abstracts to rank answers after question focus entity type filtering. Our approach retrieves relevant documents iteratively via enhanced keyword queries from a traditional search engine. To improve document retrieval performance, we introduced a supervised long short term memory neural network to select keywords from the question to facilitate iterative keyword search. Our unsupervised baseline system achieves a mean reciprocal rank score of 0.46 and Precision@1 of 0.32 on 936 questions from BioASQ. The answer sentences are further ranked by a fine-tuned bidirectional encoder representation from transformers (BERT) classifier trained using 100 answer candidate sentences per question for 492 BioASQ questions. To test ranking performance, we report a blind test on 100 questions that three independent annotators scored. These experts preferred BERT based reranking with 7% improvement on MRR and 13% improvement on Precision@1 scores on average.

## Background

While traditional information retrieval (IR) techniques employed by most search engines allow retrieval of documents deemed to be relevant to the keyword provided by a user, question answering systems provide precise answers to natural language questions. Natural language questions together with precise answers allow more elaborate, nuanced and direct inquiries into the ever expanding body of biomedical literature to guide biomedical knowledge discovery. Biomedical question answering poses additional challenges to already challenging question answering task due to its domain-specific terminologies with a plethora of ever increasing subdomain specific terminology and language style variations.

From a question to the corresponding answer(s), a question answering system mostly consists of three main processing phases involving the processing of the question, document processing that includes retrieval and selection of relevant documents, which could potentially answer the question and answer processing including answer matching, ranking and selection (1).

One of the most important requirements for the development of a question answering system is an expert generated training/evaluation question answer dataset. BioASQ, an EU-funded biomedical semantic indexing and question answering challenge (2) provides accumulated sets of biomedical question/gold standard answer data each year since the inception of the challenge in 2013. BioASQ datasets are cumulative. Every year, the test questions together with gold standard of the previous year are added to the QA corpus. BioASQ covers a large set of biomedicine subdomains including medicine/clinical questions (such as “Describe the mechanism of action of drisapersen” or “What memory problems are reported in the ‘Gulf war syndrome'?”), molecular biology and biochemistry (such as “Which SWI/SNF protein complex subunit has been demonstrated to interact with the FANCA gene product?”) and bioinformatics (such as “Which is the execution time (complexity) of the Smith-Waterman algorithm for the alignment of two sequences”). Four types of questions are provided by BioASQ; (1) “Yes/No” questions such as “Is miR-21 related to carcinogenesis?”, (2) Single factoid questions such as “which is the most common disease attributed to the malfunction or absence of primary cilia?”, (3) list factoid question such as “which human genes are more commonly related to craniosynostosis?” and (4) summary questions such as “what is the mechanism of action of abiraterone?.” We obtained the BioASQ 2017 Task 5b question answering training/development dataset for our system development and evaluation. For all the four types of questions, we have selected a sentence as our answer representation. All yes/no question answers in the BioASQ dataset were implicit in the PubMed abstract sentences. Most summary questions can be answered by a single sentence. A sentence provides the context around the factoid/list answer to interpret the validity of the answer.

In this paper, we describe a question–answering system named Bio-AnswerFinder that presents biomedical researchers with sentences from PubMed abstracts that most likely provide the answer to their question that relies, for answer ranking, mainly on the transfer of syntactic and semantic information from unsupervised language modeling instead of more traditional explicit knowledge representation. The system uses query expansion and weighted iterative keyword queries for relevant document retrieval, a weighted version of relaxed word mover’s distance (rWMD) (3) based similarity on GLoVE (4) word/phrase embeddings that is learned in an unsupervised manner from PubMed abstracts for answer sentence ranking. Sentence level semantics are transferred from language modeling by bidirectional encoder representations from transformers (BERT) (5) based deep learning language representation, which is fine-tuned for answer sentence reranking. The BERT based rerankings are preferred over the baseline weighted rWMD ranking by all three annotators on blind-method comparisons.

### Related work

Athenikos and Han (1) overview biomedical QA systems, which are semantic-, inference-, or logic-based. BioASQ challenges helped to push advances in biomedical question answering systems further by providing a large set of training data and testing data for the participants of the challenges. Various question answering systems have participated in the BioASQ challenges over the years, such as BioAMA (6) and UNCC QA (7) from the latest challenge, which had relied on named entity recognition for factoid and list questions. UNCC QA received the highest ROGUE scores for summary questions using lexical chains based extractive summarization. For yes/no questions, BioAMA used textual entailment via a hierarchical convolutional neural network (NN).

Wiese *et al*. (8) introduced a neural system to detect answer spans in the gold standard snippets provided by BioASQ for question answering task (Task B) competition. Their system was first pretrained on the SQUAD (9), a 100 000-factoid question answering dataset generated from Wikipedia abstracts by crowdsourcing. Then, they fine-tuned their system on BioASQ 5b factoid and list subset achieving state of the art performance on the factoid questions.

Relaxed word mover’s distance is used by Brokos *et al*. (10) in a *k*-nearest neighbor setting to retrieve the most similar PubMed abstracts to the question GLoVE vector. In our system , we use a weighted version of the relaxed word mover’s distance to rank sentences of abstracts returned by our iterative question to keyword query enhancer, which are further reranked by a BERT (5) based deep neural network (DNN). BioAMA uses maximum marginal relevance algorithm (11) for sentence selection, where we use weighted-relaxed word mover's distance (wRWMD) with word embeddings followed by sentence level language modeling using a fine-tuned BERT classifier. Lee *et al*. (12) further pretrained BERT with PubMed abstracts and/or PubMed Central (PMC) open access full text articles to create a domain adapted model called BioBERT and using the gold standard passages provided in BioASQ 5b dataset for factoid questions to detect contiguous words of the named entity answering the question similar to BERT (5) question answering test setting. Their system is also pretrained with SQUAD (9) similar to the QA classifier by Wiese *et al*. (8). Bio-AnswerFinder, however, is an end-to-end system that does not depend on gold standard passages to find the answer, which is not realistic for real-word applications. Rather, it retrieves candidate abstracts from a traditional search engine and uses BERT to rerank wRWMD ranked sentences covering all four forms of question types. We have compared BioBERT fine-tuned reranker with our non-domain adapted BERT reranker and found that there was no performance difference between them. We have also implemented a simple exact answer extraction module for factoid type questions to compare Bio-AnswerFinder to other state of the art QA systems.

## Materials and Methods

**Figure 1:**
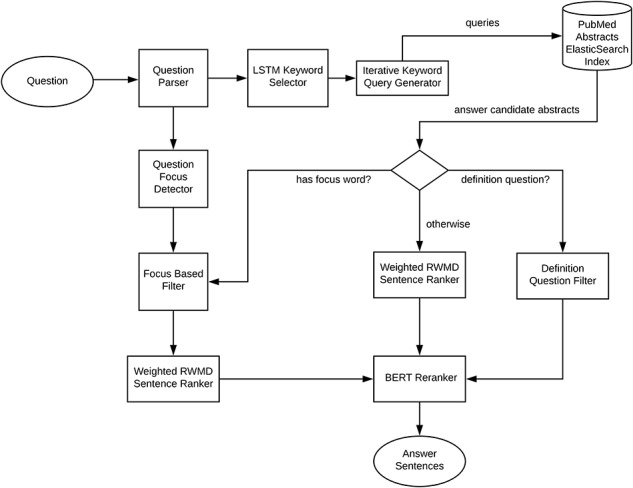
Overview of the Bio-AnswerFinder.

Our system follows the three main processing phases common to all question answering systems (1). An overview of the introduced system is shown in Figure 1. The question processing phase includes question parsing, detection of the focus of the question, supervised long short term memory (LSTM) (13) and DNN-based keyword selection, question to keyword query conversion and query expansion. The document processing phase includes an iterative most specific to most generic keyword search guided by the LSTM network selected keywords to retrieve a focused mostly relevant set of documents from an Elasticsearch index of PubMed abstracts (RRID:SCR_004846). The answer processing phase, based on the detected question type (focus, definition question or other), involves focus entity type or definition pattern based filtering of the sentences from the retrieved abstracts. For focus and other non-definition questions, the answer candidate sentences are then ranked by a weighted version of the relaxed word mover’s distance (3). The sentences are further reranked by a fine-tuned BERT (5) classifier.

For the biomedical corpus, the full set of PubMed abstracts from 2017 are ingested, transformed and indexed to an Elasticsearch endpoint using our ETL system, Foundry (14). The system uses the title and abstract text for each abstract to find answers.

The subcomponents of the Bio-AnswerFinder are detailed in the upcoming sections.

### Preprocessing

To capture semantic and syntactic relationships among words co-occurring in similar contexts in a vector space representation amenable to IR, we use GloVE (4) as our word embedding model. GloVE (4) is a vector space model to represent words combining advantages of global matrix factorization and local context window based word representation (15) methods.

For the PubMed corpus, we have considered *n*-grams of length one through four words. *N*-grams of length two through four are selected by hypothesis testing to detect phrases instead of *n*-grams co-occurring by chance. Phrase detection was done on the 300 million word corpus of abstracts extracted from the PMC open access full paper distribution (August 2017). GloVE training is applied to the combined set of single terms and phrases in the full set of PubMed (August 2017) abstracts (2.5B words) to generate 100 dimensional embedding vectors for each word/phrase occurring more than five times in the corpus resulting in a vocabulary of ~2.5 million unique words and phrases. Document frequencies are precalculated for each word/phrase to be used to weight terms in natural language query processing by inverse document frequency (IDF).

### Question processing

*Natural language question analysis*. Analysis of a question to determine what it is asking for is an essential part of question answering systems such as IBM Watson (16). The analysis of questions in our system entails the following:

*1) Question Parsing*

Natural language questions are first tokenized and then sentences (for multi-sentence) questions are detected following the part-of-speech (POS) tagging, lemmatization, constituent and dependency parsing using the Stanford CoreNLP (17) library. Contiguous noun phrases (NPs) in the question are also detected using the constituent syntax tree.

*2) Question Type Detection*

The type of a question determines the strategies to be used to answer it. Definition questions are answered by answer pattern matching (18). The entity type of the focus word/phrase of a factoid question is used to filter out answer candidates not having the correct entity type.

*3) Detection of the Focus of a Question*

To generate the appropriate keyword query to retrieve relevant documents to find the answer to the question, detection of the focus words/phrases of the question is very important. The system first detects the wh word (what, where, how etc.) in the question. The focus word/phrase is usually located either immediately after the wh word or for copula/auxiliary verbs, after the verb. For prepositional phrases using the prepositions ‘of’ and ‘in,’ the focus phrase comes after the preposition and the NP coming before is usually a modifier of the focus phrase.

*From question to keyword query/ answer candidate document retrieval.* The first step in question sentence to boolean keyword query conversion for traditional search engines like Elasticsearch is detection of non-copula/auxiliary verb(s) in the question. The verbs are morphologically converted to their infinitive forms. For all nouns and NPs their singular/plural forms are also included in the candidate search keyword set. Noun phrases are normalized by removing articles, pronouns, gerunds and possessive characters from them. Slash words such as ‘drug/medication’ are tokenized into multiple candidate search keywords. Coordinated list of terms/phrases are detected and converted into a list of candidate phrase search terms. For the detected verbs, NomLEX (19) is used to find any nominalizations as additional search terms together with its conjugated forms. The question analyzer subsystem also tries to resolve any acronyms used in the question to determine its expansion and adds the expansion to the candidate search terms. Similarly, any acronyms for a NP in the question are detected and added to the candidate search terms.

Sub-phrases within NPs are detected using the vocabulary of terms and *n*-grams generated from the corpus. Starting from both ends of a NP, words are dropped from the phrase and checked against the vocabulary to include matching subphrases as additional candidate search terms. Also each word in a question NP is also considered as a candidate search term. The candidate search terms are weighted by the IDF of each term/phrase in the vocabulary.

### Document processing

*Recognition of entities in a question and candidate answers.* The ability to detect classes of named entities in a question and potential answers is necessary to generate equivalency classes enabling generalization from a specific question to extract its intent. The scope of answerable questions from a corpus depends on the type and number of entities recognized. The entities are the most domain specific part of any QA system. In Bio-AnswerFinder, we recognize nine entity types; gene, protein, enzyme, disease, drug, molecular entity, organism, anatomical entity and cellular component. All of these entities are currently recognized by lookup. The gene lookup table is generated from the HUGO database. The disease names are extracted from the CDC Diseases & Conditions website. The drug lookup table is generated from DrugBank (RRID:SCR_002700) data in Scicrunch.org (20) and the rest from Scicrunch biomedical ontologies.

*Iterative Elasticsearch keyword queries to retrieve Answer Candidate Documents.* Inspired by the iterative document retrieval approach of Pasca (18), we generate a set of keyword search queries of decreasing complexity to retrieve a set of relevant documents to locate the answer supporting sentence in them. From the candidate search term information, a sequence of keyword search queries are generated against the PubMed abstracts Elasticsearch index. The keyword query start as an AND query with all candidates and expansions included and keywords are excluded until some results are returned but the result set is not very large (>1000) (indicating a too generic query). The keyword query consists of NPs disjuncted with their singular/plural form and acronyms and verbs disjuncted together with their nominalizations, infinitives and conjugations. Each disjunction group is then combined with logical AND constructs together with subphrases and individual words of NPs in the question to form the keyword search query for Elasticsearch. All the terms/phrases within each search construct are sorted by their weights in descending order. The weight of a logical disjunctional search construct is calculated by the following formula from its constituent term and phrase weights;

$$ {w}_{\mathrm{cons}}=\frac{1}{\left|\mathrm{ST}\right|}\left[\sum_{i\in V}{w}_i+\sum_{i\notin V}\underset{j}{\min\ }{w}_{ij}\right] $$

where $|\mathrm{ST}|$ is the number of search terms in a search construct, *V* is the vocabulary set, $i=1,\dots, |\mathrm{ST}|$and $j=1,\dots, \#\mathrm{of}\ \mathrm{tokens}\ \mathrm{in}\ {\mathrm{ST}}_i$. The search terms are dropped from each disjunctional search construct one at a time in increasing weight order until any matching documents are retrieved.

The iterative process is illustrated on the following BioASQ question;

“Is alemtuzumab effective for remission induction in patients diagnosed with T-cell prolymphocytic leukemia?”

**Figure 2:**
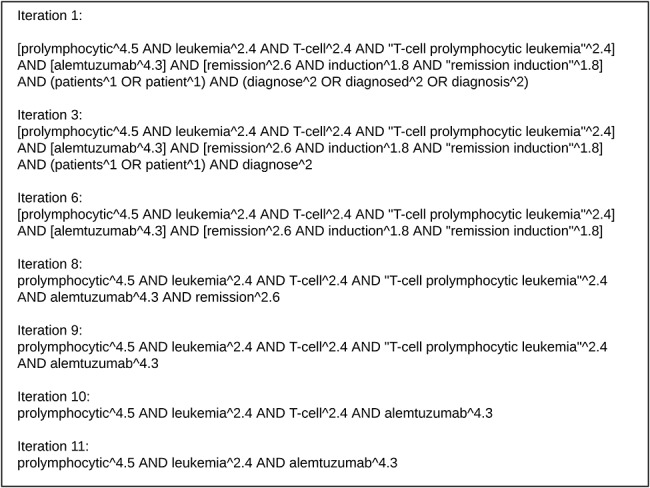
Illustration of iterative weighted Elasticsearch keyword querying process on the question “Is alemtuzumab effective for remission induction in patients diagnosed with T-cell prolymphocytic leukemia?”

Here all the nouns, NPs and verb ‘diagnose’ are considered for keyword query generation. Each term and phrase is weighted by the formula given above and weights are normalized. Figure 2 depicts the keyword query sent to our Elasticsearch index with additional square brackets to show search constructs considered as a unit. During greedy iterative generalization of the keyword query, the term with the lowest weight from each unit were dropped one at a time until some abstracts are returned. The process starts with the group having the least maximum weight among its elements. The verb search construct(s) are always considered first in the dropping process regardless of their weights. Also any entities (drug, disease etc.) recognized are treated specially. Since they usually indicate the focus of the question, the dropping process tries to leave them in the query and continue to drop terms from other search constructs, which have more than one term and come before those entities in the query. This is witnessed in iterations 9 through 11 shown in Figure 2 for the drug alemtuzumab. Once all search constructs are reduced to a single term/phrase, then focus entities are considered for dropping, as the last resort.

### Answer processing

*Candidate answer sentence ranking by weighted Relaxed Word Mover’s Distance*. To select the most likely sentences in the retrieved candidate documents, we need a way to rank them. This ranking can be done in a supervised manner or by unsupervised means relying on similarity between the question and candidate answer sentence. Kusner *et al*. (3) cast the semantic similarity between documents as a transportation problem, where the dissimilarity between two documents is considered as the minimum distance that the embedded word vectors of one document need to travel to reach the embedded word vectors of the other document. By analogy to the earth mover’s distance transportation problem, they introduced word mover’s distance (WMD), a constrained optimization problem.

Given several suppliers each with fixed amount goods and several consumers each with limited capacity together with the transportation cost between each supplier-consumer pair, the transportation problem can be defined as the cheapest way of distributing the goods to the consumers. Applied to the semantic similarity between two documents modeled with a set of word embeddings (using bag or words assumption) *d* and *d*’, transportation cost *c*(*i*,*j*) between word *i* from *d* and word *j* from *d*’ is the Euclidean distance between word embedding vectors *v_i_* and *v_j_*. The amount of goods available per supplier (word) of the document *i* is its normalized document frequency *d_i_*. Similarly, the capacity per consumer (word) of the document *j* is also its normalized document frequency *d_i_*. Defining the fraction of goods sent from word *i* of document *d* to the word *j* of document *d*^’^ as *T_ij_*, the transportation problem for WMD can be stated by the following linear program;

$$ \underset{T\ge 0}{\min}\sum_{i,j=1}^n{T}_{ij}c\left(i,j\right) $$

$$ \mathrm{subject}\ \mathrm{to}:\sum_j{T}_{ij}={d}_i\ {\forall}_i\in \left\{1,\dots, n\right\} $$

$$ \qquad\qquad\quad\sum_i{T}_{ij}={d}_j\ {\forall}_j\in \left\{1,\dots, n\right\} $$

where *n* is the size of the vocabulary. Relaxing the constrained optimization problem by dropping one of the constraints results in a lower bound to the full optimization problem with *O*(*p*^2^) complexity, where *p* is the number of unique words in the documents compared. If the second constraint is removed, the optimal solution is obtained if for each word in *d* (question) all the normalized document frequency is moved to the most similar word in the document *d*’ (answer candidate). We found out experimentally that IDF-based weighting of terms improves vanilla relaxed WMD. Thus, the weighted relaxed WMD for a question–answer sentence pair becomes

$$ \mathrm{wRWMD}\left(Q,A\right)=\frac{\sum_{i\in{I}_Q}{w}_i\times \underset{j\in{I}_A}{\max}\ \mathrm{cossim}\left({v}_i,{v}_j\right)}{\sum_{k\in{I}_q}{w}_k} $$

where ${I}_Q$ and ${I}_A$ are index sets for the unique terms/words in question *Q* and candidate answer sentence *A*, respectively. The semantic vector for word $i$ is noted as ${v}_i$ and each word $i$ is weighted by ${w}_i$(normalized IDF).

The candidate documents returned from the Elasticsearch index are first segmented into sentences using the Stanford CoreNLP and wRWMD is calculated for each answer candidate sentence. Sentences are then ordered by descending similarity (1 – wRWMD) order.

*Candidate answer filtering for question focus entity type to answer factoid questions.* If the focus of the question matches any of the entity types recognized, then the answer must contain at least one entity of the same type to be considered an answer of the asked focus entity type. Our system detects entities in the candidate answer sentences and corresponding question and filters out any sentences not having any entity of the focus entity type.

*Supervised answer candidate reranking.* For supervised reranking of the candidate answer sentences, we have used a DNN-based language representation model named BERT (5). BERT is based only on attention based feedforward NN layers unlike the more common complex recurrent and convolutional NN based encoder/decoder architectures used for language transduction models allowing for better parallelization and much shorter training times (21). The pretrained BERT models and code were open sourced by Google on GitHub (https://github.com/google-research/bert). Pretrained BERT models are then combined with additional classifier layers and fine tuned for different NLP tasks achieving state of the art performance in each task (5).

We have cast answer candidate reranking as a sentence pair classification task to decide if an answer candidate sentence contains the answer for the question given a question and answer candidate sentence pair. We have used the ${\mathrm{BERT}}_{\mathrm{BASE}}$ pretrained model with 110M parameters. Using maximum sequence length of 64 and batch size of 16, the pretrained model fits the memory of our GPU (GTX 1060 6GB) and was fine tuned for three epochs in about an hour. For training/testing data, we have used 592 questions having an answer in the first 100 results returned by our baseline unsupervised wRWMD based QA system. Since we have annotated the results for the first occurrence of an answer in the answer candidate list for each question, there could be other potential answers below the first occurrence making our training data noisy. The probabilities from the binary classification task are then used to rerank the answer candidates. The BERT and wRWMD based reranking was then compared in a blinded evaluation study, where three annotators presented with the first 10 ranked answer candidates for each method for each question in the test set.

*Exact answer phrase extraction for factoid questions.* In BioASQ, the exact answer for a factoid question, in majority of the cases, is either a single term or a phrase. The state of the art supervised QA systems (8, 12) detect the start and end positions of the exact answer in the BioASQ provided gold standard snippets for factoid questions. About 30% of the BioASQ factoid questions cannot be answered by this extractive approach since the answer tokens are not available in the snippets (12, 22).

To provide exact answers for factoid questions in Bio-AnswerFinder, we introduced a simple method based on the focus word detection subsystem. The first 10 answer candidate sentences selected by the Bio-AnswerFinder were considered for exact answer candidate selection. If the detected focus term is one of the nine recognized named entity types, all the unique entities of the recognized type in the answer candidate sentences are returned in their first occurrence order in these sentences as the exact answer candidates.

For the focus terms that are not a recognized entity type, all of the noun phrases are potential exact answer candidates. Any determiners, demonstrative pronouns, predeterminers, comparative or superlative adjectives, cardinal numbers and adverbs were stripped from the beginning of the noun phrases extracted from CoreNLP parsed sentences. For NPs of three to five tokens subphrases are also included in the exact answer candidate list.

Using GloVE word/phrase embeddings, the cosine similarity of each exact answer candidate NP to the focus term GloVE vector was calculated for ranking purposes. Since the focus term denotes the type of the answer for a factoid question, we expect that it should be similar to the answer, usually, by a subsumption relation. Word embeddings, however, encode other syntactic and semantic aspects of the similarity besides subsumption. We tried to minimize the effect of these additional aspects of the word embeddings based similarity, by two ways of systematic filtering. First, any non-informative named entity terms such as “protein,” “gene,” “drug” or “disease” occurring in the candidate set by themselves were removed from the list to minimize the noise. Second, under the assumption that the question does not contain its answer, any candidate NPs which were very similar to any noun/NP (based on the GloVE vector cosine similarity having ≥0.8) in the question were filtered out. The cosine similarities of the candidate NPs were further weighted by their TF-IDF score with the term frequencies determined from the candidate sentences. These scores were then used to rank non-entity focus term generated exact answer candidates in decreasing score order.

## Results and Discussion

### Evaluation

To evaluate the performance of the introduced QA system, we have used mean reciprocal rank (MRR) score and Precision@1, which are commonly used to evaluate QA system performance (23, 2). MRR is calculated as

$$ \mathrm{MRR}=\frac{1}{N}\sum_{i=1}^N\frac{1}{\operatorname{rank}\left({Q}_i\right)} $$

where $N$ is the number of questions and $\operatorname{rank}\Big({Q}_i\Big)$ is the rank order of the topmost correct answer for question *i*. Precision@1 is defined as the ratio of the number of questions answered by the first returned answer candidate to the total number of questions.

Besides the question keyword and answer reranking components, our system does not need any supervised training data. The answer reranking DNN relies on the supervised data from manually selected answers from the weighted RWMD ranked sentences. In order to both evaluate the unsupervised (baseline) version of our system and to generate training data for the reranking classifier, we used the BioASQ question–answer training corpora 5b which has 1799 questions, out of which 1114 questions–answer pairs had exact answer and ideal answer sentences from abstracts, which we can automatically align with our PubMed abstracts Elasticsearch index generated using our Foundry ETL system (12). Out of these 1114 question–answer pairs, we selected 85% (936 unique question–answer pairs) for performance analysis. Due to ongoing scientific research, new papers/abstracts are constantly added to the PubMed abstract corpus. A question can be answered by new abstracts or different abstracts than the ones the BioASQ curators have chosen at the time of annotation. For this reason, performances of the participants in the QA section of the BioASQ annual competition are also manually inspected. Using the exact and ideal answer gold standard BioASQ texts as a guideline, we have manually selected the first occurrence of the answer in the first 200 sentences returned by our baseline system on the 936 questions. For list questions, a sentence containing at least one answer item from the list is considered as a correct answer. Out of 936 questions, 595 questions had been answered (based on exact and ideal answer text provided) by a sentence from an abstract not annotated by the BioASQ curators. For our unsupervised baseline system, the MRR on these 936 questions was 0.46 and the Precision@1 was 0.32.

### Datasets for supervised classifiers

For the supervised keyword selection and answer candidate reranking, we used the 936 unique questions for the evaluation of our unsupervised baseline question answering system.

For supervised answer reranking we selected all the questions having an answer in the first 100 answer candidates based on the baseline system evaluation resulting in 592 questions and answer candidate set pairs. The threshold 100 is selected to not have an overly unbalanced training dataset (one correct, 99 incorrect). We have randomly hold out 100 questions out of this set as the testing set.

### Supervised keyword selection

For the keyword selection classification task, we have tried NN architectures with PubMed abstract corpus trained GLoVE word embeddings encoded into a fixed length representation that is fed into a dense layer of fixed length sigmoid neurons. Each question is tokenized and for each token the corresponding GLoVE word embedding vector is looked up and concatenated into a fixed length sequence (40 in our case based on max BioASQ tokens of 23) as the NN input beyond which we truncate the remaining tokens. Shorter sequences are zero padded. The number of output neurons is also fixed to 40, one for each token indicating whether that token is a keyword or not. As for the encoder layer, we have tried a dense layer, a LSTM (13) layer and an one-dimensional convolutional NN layer. As a second NN architecture, we also tried a multi-input architecture where beyond the GLoVE word embeddings POS tags are encoded by a dense rectifier linear unit layer before being concatenated to the word embedding encoder layer results and connected to the sigmoid output layer. The intuition behind the second architecture is based on the supposition that nouns are best candidates for good keywords, thus POS information might inform the keyword selection process.

The models are trained in an 8-fold cross-validation setting, where one eighth of the training set is randomly held out without replacement for performance testing for each fold. The DNN models are implemented using the Keras (https://keras.io) deep-learning framework. The results (average precision, recall and F1 values together with their standard deviations) are summarized in Table 1. For each model, its complexity in terms of number of trainable weights and dropout regularization probability is also shown. The significance of performance differences are assessed by two-tailed *t*-tests. The baseline LSTM model significantly outperforms both baseline dense model (*P*-value = 9.3e-07) and baseline convolutional NN (*P*-value = 0.0001).

**Table 1:** Keyword selection classifier model performances

**Model**	**Precision (SD)**	**Recall (SD)**	**F1 (SD)**
Baseline dense model, dropout = 0.2, 161 680 parameters	84.4 (2.1)	77.3 (2.3)	80.7 (2.3)
Baseline LSTM model, dropout = 0.2, 86 600 parameters	89.7 (2.0)	88.7 (2.4)	89.2 (1.4)^*^
Baseline ConvNet model, 44 120 parameters	84.8 (1.8)	82.8 (5.3)	83.6 (2.4)
Multi-input models			
Dense–dense model, dropout = 0.2, 177 290 parameters	86.6 (2.7)	79.9 (3.2)	83.1 (2.6)
LSTM-dense model, dropout = 0.2, 102 210 parameters	89.5 (1.9)	89.6 (1.5)	89.5 (1.4)
LSTM–LSTM model, dropout = 0.2, 104 560 parameters	91.2 (1.3)	88.1 (2.2)	89.6 (1.4)

Asterisk indicates statistical significance.

The performance improvements of the multi-input dense-LSTM and LSTM–LSTM models over the baseline LSTM model were not statistically significant.

### Effect of keyword selector classifier on retrieval

To see the effect of keyword selection on the final QA system, together with additional iterative document retrieval approaches (24), we have selected 100 questions randomly from the set of the most difficult questions, as determined by the result analysis on the wRWMD only baseline system as the test set. The full system is run first without the inclusion of keywords selected (baseline) and then with the keywords, selected by the classifier, ensured to be included in the initial query submitted to the search engine. The first 10 results returned for each test question is then checked by a curator to determine the rank of the first answer if any. The results shown in Table 2, indicates that keyword selection increases both MRR and Precision@1. Using keyword selection together with supervised keyword ranking for greedy iterative query term dropping further improved overall QA performance more than doubling the MRR to 0.39 and almost doubling the Precision@1 score to 0.32 on this most challenging subset of the BioASQ 5b dataset (24).

**Table 2:** Effect of supervised keyword selection on the end-to-end QA performance

**Retrieval method**	**MRR**	**Precision@1**
Baseline	0.18	0.13
Baseline + keyword inclusion	0.21	0.15

### Blind evaluation of answer ranking methods

Weighed RWMD and BERT ranked results were assessed by three curators (experts from related scientific fields) independently and blindly as to the method. Each curator was presented with a question, and a set of 10 answer candidates per ranking method, though it was hidden from the curator which method was being presented (results are summarized in Table 3). The curators were instructed to choose the first correct answer to the question, if any correct answer was available. Also available to curators were the gold standard answer(s) and the snippets from BioASQ 5b dataset.

All curators agreed on the same answer for 55 out of 100 questions with wRWMD and 53 with BERT ranked method. These cases were not examined further, however, we considered the cases where at least one curator disagreed with the others by examining the sentences with an additional fourth curator to determine the causes of the disagreement.

**Table 3:** Answer selection blinded method performance evaluations

	**Weighted rWMD**		**BERT reranked**	
**Curators**	**MRR**	**Precision@1**	**MRR**	**Precision@1**
Curator 1	0.747	0.61	0.813	0.71
Curator 2	0.619	0.49	0.657	0.56
Curator 3	0.743	0.60	0.781	0.66

The most common type of disagreement was paraphrasing, i.e. where both answers selected are correct but the phrasing is different and may be preferred by one curator or another.

The curator disagreement varied based on the type of question as shown in Table 4. The factoid questions had the least amount of disagreement and highest performance, while the summary type questions having the largest disagreement. Summary questions are best answered by a multiple sentence explanation. It is usually not possible to summarize all the aspects of the required answer in a single sentence. Thus, the curators needed to select the sentence that had the most important aspect of the answer which is highly subjective.

**Table 4:** Answer selection performance based on question type

**Question type (size)**	**Annotator 1 (MRR)**	**Annotator 1 (Prec@1)**	**Annotator 2 (MRR)**	**Annotator 2 (Prec@1)**	**Annotator 3 (MRR)**	**Annotator 3 (Prec@1)**
BERT reranking						
Summary (14)	0.895	0.86	0.495	0.43	0.661	0.57
Yesno (23)	0.737	0.70	0.688	0.57	0.779	0.65
Factoid (35)	0.867	0.77	0.819	0.74	0.884	0.80
List (28)	0.725	0.57	0.510	0.39	0.715	0.54
wRWMD ranking						
Summary (14)	0.723	0.57	0.560	0.43	0.812	0.71
Yesno (23)	0.809	0.65	0.708	0.61	0.741	0.57
Factoid (35)	0.790	0.69	0.705	0.57	0.799	0.69
List (28)	0.654	0.50	0.469	0.32	0.639	0.46

To examine the effect of domain adaptation to reranking, we have obtained BioBERT v1.1 model further pretrained with PubMed abstracts from GitHub (https://github.com/naver/biobert-pretrained/releases/tag/v1.1-pubmed; RRID:SCR_017547) and used it to fine-tune a second reranker similar to our BERT fine-tuned reranker. The effect for BioBERT domain adaptation to reranking is examined by using the BioBERT fine-tuned reranker classifier to classify the first 100 results from wRWMD for the same 100 test questions as in the BERT_BASE_ case. Then, these 100 wRWMD sentences per test question were sorted by the decreasing BioBERT reranker score for reranking. Afterward, the 10 sentences with highest BioBERT reranker score for each question were checked by one curator against the gold standard and curated results from BERT_BASE_ fine-tuned reranker. Using the answers from the three curators and the BioASQ gold standard answers on the 100 test questions, we have calculated the MRR and Precision@1 scores for both rerankers. Using majority vote among curator answers together with BioASQ gold standard answer double checking, BERT reranker had MRR of 0.727 and Precision@1 of 0.62 and BioBERT reranker had virtually identical MRR of 0.723 and Precision@1 of 0.62.

Out of 100 questions BERT and BioBERT rerankers agree on 61 questions. The most common agreement was at the first rank (on 50 questions) followed by no answer (on seven questions). On the remaining 39 questions, BERT reranker had better ranking over the BioBERT reranker for 20 questions and BioBERT reranker had better ranking over the BERT reranker on the remaining 19 questions. Out of the 50 first rank agreement questions, BioBERT and BERT rerankers selected different paraphrasing in 34 questions. Even though the ranking was the same for both methods, in significant number of cases the paraphrasing selected by BioBERT was qualitatively better than BERT ranking.

For the question “Which is the most abundant membrane protein on Earth?,” as an example, BERT reranker selected the sentence “Degradation of the most abundant membrane protein on earth, the light-harvesting complex of Photosystem II (LHC II), is highly regulated under various environmental conditions, e.g. light stress, to prevent photochemical damage to the reaction center.” While the BioBERT reranker selected the more direct answer sentence “LHCII is the most abundant membrane protein on earth.”.

Seeing virtually no difference between BERT and BioBERT for reranking task could be explained by the following observations; BERT was trained on Wikipedia data which already contains a substantial amount of information about biomedical topics. It was trained to learn word part (by using WordPiece tokenization (25)) level embeddings instead of more traditional word based embeddings facilitating representation of embeddings for the out-of-vocabulary words by combination of word part embeddings minimizing the need for domain adaptation to provide domain specific vocabulary information. The contextualized representations learned by BERT were recently shown to embed dependency syntax parse tree information (26). This rich syntax information, together with the domain-specific GloVE word vectors-based wRWMD ranking, which provides the input to the BERT reranking, might be enough for the reranking task. The wRWMD being a strong baseline even before BERT reranking provides evidence to this. Also BERT rerankings had strong Precision@1 values indicating that substantial amount of questions is answered by the first returned sentence, which cannot be further improved.

Bio-AnswerFinder is designed and implemented with the goal of being the next generation literature search interface for the Scicrunch.org platform (https://scicrunch.org), which primarily serves the biomedical research community. In their quest for knowledge discovery, biomedical researchers require evidence about the exact answer returned. We have decided to present sentences supporting the answer instead of the exact answer(s) extracted from ranked sentences as our output to provide the context for the exact answer. None of the question answering systems has perfect precision and recall and without this guarantee the returned answer needs to be verified by the user by evaluating the supporting evidence.

### Comparison to other QA systems

While Bio-AnswerFinder is an end-to-end (from the question to the answer sentence) system, the best performing biomedical QA systems use a narrower definition of question answering namely finding the spans of tokens answering the question in the set of gold standard snippets.

Having gold standard snippets to find answers is not realistic for a production QA system to be used by biomedical researchers. Also extractive supervised question answering as used by Wiese *et al*. (6) and BioBERT (12), limits the question type to be answered to factoid questions. To compare Bio-AnswerFinder with these QA systems, we used a simple, unsupervised noun phrase extraction and ranking approach based on the question focus word type and similarity as described in section “Exact answer phrase extraction for factoid questions.” We applied this approach to the first 10 sentences returned by the Bio-AnswerFinder with BERT reranker for the test set curated by the curators. Also, to get a lower bound on the MRR performance, we used the challenging test set from our document retrieval approach comparison study (24). Using the keyword importance classifier based retrieval approach (24) as the retrieval system in the Bio-AnswerFinder, the exact answer candidates are extracted from the first ten answer sentences per factoid test question.

The exact answer MRR results together with the sentence level curator results are shown in Table 5. Bio-AnswerFinder showed performance close to the second best system (8) at the exact answer level. Taking into account that exact answer extraction was unsupervised while all other approaches were supervised and no gold standard snippets were used unlike the other systems, this is encouraging. Also, at the sentence level, Bio-AnswerFinder outperformed the state of the art (12) even for the challenging test set. The performance difference at the exact answer versus at the sentence level for Bio-AnswerFinder can be partially explained by the shortcomings of the simple unsupervised approach used not being able to filter all irrelevant phrases. Also, exact answer phrase selection is a more difficult task than the sentence selection, since the search space for exact answer phrases is much larger than the search space for sentences where each sentence usually yielding ~5 to 10 unique candidate phrases to be considered. However, sentence level answer representation together with definition question processing capabilities of Bio-AnswerFinder allowed answering wider range of factoid questions which are not amendable for extractive question answering since the answer does occur in paraphrased or sentence format. About 30% of the BioASQ factoid questions fall into this category (12, 22). Thus, sentence level answer representation allowed wider range of factoid questions to be answered while also providing supporting context for the answer.

**Table 5:** Factoid question exact answer comparison with other QA systems

**QA system**	**MRR for factoid questions**
BioAMA (6)	0.195
Wiese *et al*. (8) (average of five batches)	0.405
BioBERT (12)	0.483
Bio-AnswerFinder (challenging set (24))	0.239 (0.508 at the sentence level)
Bio-AnswerFinder (average of three curators)	0.381 (0.857 at the sentence level)

## Conclusion

In this article, we introduced a question answering system to aid biomedical researchers in their knowledge discovery efforts. The system relies on language modeling for words/phrases and whole sentences (in case of BERT) learned in an unsupervised manner from large corpora for implicit semantic information used in answer selection. Using an iterative document retrieval process, enhanced by DNN-based keyword selection, relevant documents to find answer(s) for the question are retrieved. The candidate documents retrieved for non-definition questions for which the focus of the question is detected are filtered by a focus type filter. Blind tests by three curators provided evidence that BERT based reranking of the weighted relaxed WMD ranked answer candidate sentences further improved the performance of the system especially for the Precision@1. Current work is focused on integrating the system into the SciCrunch (20) framework as the next generation biomedical search engine after more tests on biomedical question answering and system optimizations.

## Ethics approval and consent to participate

Not applicable.

## Consent for publication

Not applicable.

## Availability of data and materials

Bio-AnswerFinder source code and documentation together is available on GitHub (https://github.com/scicrunch/bio-answerfinder). The datasets generated during and/or analyzed during the current study are available in the Zenodo repository, doi: 10.5281/zenodo.2597595.

## Authors’ contributions

I.B.O. designed and implemented the introduced system and conducted the experiments on the system, and was a major contributor in writing the manuscript. A.B. together with I.B.O. did the analysis of the curator results on the blind reranking method tests and was a major contributor in writing the manuscript. J.S.G. was a major contributor in writing the manuscript. All authors read and approved the final manuscript.

## Notes

**Database URL**: https://github.com/SciCrunch/bio-answerfinder
